# Gut Microbiota Profiles Differ among Individuals Depending on Their Region of Origin: An Italian Pilot Study

**DOI:** 10.3390/ijerph16214065

**Published:** 2019-10-23

**Authors:** Andrea Fontana, Concetta Panebianco, Andrea Picchianti-Diamanti, Bruno Laganà, Duccio Cavalieri, Adele Potenza, Riccardo Pracella, Elena Binda, Massimiliano Copetti, Valerio Pazienza

**Affiliations:** 1 Unit of Biostatistics, Fondazione IRCCS Casa Sollievo della Sofferenza, 71013 San Giovanni Rotondo, Italy; a.fontana@operapadrepio.it (A.F.); m.copetti@operapadrepio.it (M.C.); 2 Division of Gastroenterology, Fondazione IRCCS Casa Sollievo della Sofferenza, 71013 San Giovanni Rotondo, Italy; panebianco.c@gmail.com; 3 Department of Clinical and Molecular Medicine, Sant’Andrea University Hospital, Sapienza University of Rome, 00185 Rome, Italy; andrea.picchiantidiamanti@uniroma1.it (A.P.-D.); bruno.lagana@uniroma1.it (B.L.); 4 Department of Biology, University of Florence, 50019 Florence, Italy; duccio.cavalieri@unifi.it; 5 Dietetic and Clinical Nutrition Unit, Fondazione IRCCS Casa Sollievo della Sofferenza, 71013 San Giovanni Rotondo, Italy; a.potenza@operapadrepio.it; 6 Cancer Stem Cells Unit, Fondazione IRCCS Casa Sollievo della Sofferenza, 71013 San Giovanni Rotondo, Italy; r.pracella@operapadrepio.it (R.P.); e.binda@operapadrepio.it (E.B.)

**Keywords:** microbiota, eubiosis, dysbiosis, geographical location

## Abstract

Background and aims: Microbiota heterogeneity among humans is mainly due to genetic background, age, dietary habits, lifestyle and local environments. In this study we investigated whether the gut microbiota profile of Italian healthy volunteers could differ based on their geographical origin. Materials and Methods: 16S rRNA gene sequencing was employed to analyze the gut microbiota of 31 healthy volunteers from three different Italian regions: Apulia (South), Lazio (Center) and Lombardy (North). Results: Differences in microbiota composition were detected when the study participants were grouped by their region of origin and when they were classified based on age classes (*p*-values < 0.05). Also species richness was significantly different both according to Italian Regions (median richness: 177.8 vs. 140.7 vs. 168.0 in Apulia, Lazio and Lombardy; *p* < 0.001) and according to age classes (median richness: 140.1 vs. 177.8 vs. 160.0 in subjects < 32, 32–41 and > 41 years; *p* < 0.001), whereas the Shannon index and beta diversity did not change. Conclusions: This study identified differences in the gut microbiota composition and richness among individuals with the same ethnicity coming from three different Italian regions. Our results underline the importance of studies on population-specific variations in human microbiota composition leading to geographically tailored approaches to microbiota engineering.

## 1. Introduction

The human microbiota with its 10^14^ symbiotic and pathogen microorganisms living within host’s body, mostly (99%) in the gut [1], of almost 1.8 kg in weight, was considered the “forgotten” or “hidden” organ [2] due to its involvement in several physiological and pathological processes [3]. Although one third of our gut microbiota is in common with most of the people, the remaining two thirds is specific to each individual as its composition is rapidly and profoundly modulated by the diet, by host genotype, lifestyle, antibiotic and/or drugs use and by local environment [4,5]. Scientists are initiating numerous microbiota projects worldwide aimed to identify the presence of a ‘core gut microbiota’, within all individuals, that could be referred as “healthy profile” or “eubiotic state” [6,7] as opposed to gut microbiota “dysbiosis” which is associated with several non-communicable diseases [8,9]. Gut microbiota eubiosis is commonly considered when potentially beneficial species belonging mainly to the two bacterial phyla *Firmicutes* and *Bacteroidetes* are found in higher and well balanced percentage while potentially pathogenic species, such as some of those belonging to the phylum *Proteobacteria* are present in a minor percentage [10]. Hence, several studies were performed with the intent to decipher the content, diversity and function of the microbial gut community which has an impact on human health and well-being. Using data from several nations and continents, it was previously demonstrated the existence of three main enterotypes of the gut microbiota that vary in species and functional composition [11] and that they might allow classification of human groups that could respond differently to diet or drug therapy. Innovations in high-throughput next generation sequencing technologies have updated our knowledge of the gut microbiota revealing significant heterogeneity of the gut microbiota between individuals depending on the genetic background, gender, age, local environments, dietary habits, lifestyle and many other factors [12,13,14,15]. However, up to date, it is still not clear which factor plays a major role in modeling the microbiota content. Previous studies uncovered remarkable differences in microbiota composition depending on the geographical origin, comparing populations across broad international geographical areas, often living in different socio-economic and cultural contexts [16,17,18,19]. Whether a variability exists even within small-scale geographical regions has remained substantially unexplored. A report by Shin et al. identified significant changes in gut microbiota of elderly women living either in island or inland areas of South Korea, however these two groups differ from each other for lifestyle, dietary patterns and physical activities [14]. Similarly, the differences in microbiota composition observed between Russian urban and rural populations were likely due to diverse diets, lifestyle and environment [20]. Only recently a study by He et al., analyzing 7009 individuals homogeneous for ethnicity living in 14 random districts of the Chinese province of Guangdong, revealed that the gut microbiota composition and its relationship with metabolic disease was strongly dependent on the geography, with associations found in a district not applicable to other districts [21]. Given the increasing role that the microbiota is taking as a modulator of a number of pathological conditions and their response to treatments [22,23,24,25,26], studying the population-specific variations in its composition suggests for geographically tailored community approaches to microbiota engineering which may have a potential benefits in enhancing existing diagnostic and therapeutic strategies. In the light of these considerations, our study aimed to investigate whether the gut microbiota profile of healthy volunteers from three different Italian regions, namely Apulia (South of Italy), Lazio (Center of Italy) and Lombardy (North of Italy), relatively homogeneous as concerns physiological characteristics and lifestyle, could differ based on their geographical origin. Although we are aware of the limited number of our samples we intent to design a pilot study trialing a new procedure intended for use in a larger programme of research.

## 2. Materials and Methods

### 2.1. Study Population

Thirty-one healthy control subjects were recruited at the outpatient Division of Immunology and Rheumatology, S. Andrea Hospital, Sapienza University of Rome [27], and at IRCCS “Casa Sollievo della Sofferenza” Hospital, with their respective Ethical committee approval numbers (N43/201 and N175/CE) and followed the same pre-analytical and analytical procedures, including samples collection and storage. All the subjects agreed to participate according to the ethical guidelines of the 2013 Declaration of Helsinki and signed an informed consent. Any subject under antibiotics or drugs treatment, consuming probiotics, or having a known history of inflammatory bowel disease or other autoimmune diseases were excluded. Only Caucasian as representative of the Italian population were recruited. Information regarding subject’s clinical variables (i.e., age, gender and BMI) as well as dietary, lifestyle and smoking habits were assessed the same day of the stool sample collection.

### 2.2. Sample Collection and DNA Extraction

Each participant provided the fresh stool sample in a collection tube filled with a DNA stabilization buffer (Canvax Biotech). The recruitment period spanned from 15th January 2018 to 10th May 2018. Since different DNA extraction/collection protocols can result in different diversity profiles due to variable cell resistance to common lysis methods, in order to reduce any possible bias, pre-analytical and analytical procedures, including samples storage and extraction was performed in only one center. DNA microbial extraction was performed using the QIAamp DNA Stool Mini Kit (Qiagen, Milan, Italy) starting from 250 µL of each sample according to the manufacturer’s protocol. After assessing DNA concentration and purity, samples were stored at −80° until processing.

### 2.3. Next-Generation Sequencing of Bacterial 16S rRNA Gene

The V3–V4 hypervariable region of the bacterial 16S rRNA gene was amplified from total DNA according to the Illumina 16S Metagenomic Sequencing Library Preparation instructions, as previously described [27]. In summary, the V3–V4 amplicon was obtained by PCR with universal primers, followed by purification. A second PCR was then performed to barcode the libraries using the Illumina dual-index system. Following a second step of purification, libraries were diluted to 4nM and pooled. Paired-end sequencing (2 × 300 cycles) was carried out on an Illumina MiSeq device (Illumina Inc., San Diego, CA, USA) according to the manufacturer’s specifications.

### 2.4. Bioinformatic Analysis

Sequence data generated as FASTQ files, deposited in the Arrayexpress repository under accession code E-MTAB-8136, were analyzed using the 16S Metagenomics GAIA 2.0 software (http://www.metagenomics.cloud, Sequentia Biotech, Barcelona, Spain, 2017; Benchmark of Gaia 2.0 using published datasets available online at: http://gaia.sequentiabiotech.com/benchmark) which performs the quality control of the reads/pairs (i.e., trimming, clipping and adapter removal steps) through FastQC and BBDuk. The reads/pairs are mapped with BWA-MEM against the custom databases (based on NCBI). The average number of reads per samples was 203,516.6 (SD +/− 81922). Rarefaction curves indicate that an adequate sequence depth was achieved (Supplementary Figure S1). For each sample, the software provided the calculation of the relative abundance of bacterial taxa, as well as the Shannon alpha diversity index, the Chao1 richness estimator and the Bray-Curtis beta-diversity index at the species level. Phylogenetic Investigation of Communities by Reconstruction of Unobserved States (PICRUSt) analysis to predict functional profiles of the three microbial communities (grouped by region) based on 16S rRNA sequencing data was performed [28]. Predicted functions were categorized at Kyoto Encyclopedia of Genes and Genome orthology (KEGG) level 2. One-way ANOVA test was performed to assess significant differences in functional categories among the three regions.

### 2.5. Statistical Analysis

Participants’ characteristics were reported as mean ± standard deviation or absolute frequency and percentages for continuous and categorical variables, respectively. For each continuous variable, the assumption of normality distribution was checked by means of Q-Q plots and Shapiro-Wilks test. For skewed continuous variables, medians along with interquartile range (i.e., IQR, first-third quartiles) were reported instead of means and non-parametric tests were performed. Overall comparisons between groups were assessed using ANOVA models (or Kruskal-Wallis test as appropriate) or Fisher exact test for continuous and categorical variables, respectively. The participants of the study were divided into three age classes with equal size (i.e., tertiles) (<32 years, between 32 and 41 years, and >41 years, respectively) and the relative abundances of microbial taxa were compared among region of origin, age classes and other lifestyle factors. The presence of a linear trend between the subject’s age (as continuous variable) and relative abundance was estimated by Spearman correlation coefficient. *p*-values from all statistical tests were adjusted for multiple comparisons, within each taxonomic level, controlling the False Discovery Rate (FDR) at level 0.05, using Benjamini-Hochberg step-up procedure. Since participants’ age distributions were not similar among regions (i.e., subjects in Lazio were significantly younger than the others), evidence of statistically differences among regions can be wrongly inferred. To this purpose, all those abundances of microbial taxa which were significantly associated with the region of origin were considered as “spurious results” if they also were significantly associated with subject’s age classes. If found, all spurious results were discarded. In order to evaluate the species richness and diversity of the microbial communities in the fecal samples of the study participants, the Chao1 and the Shannon indices were calculated for each sample, respectively. Moreover, to measure the inter-individual dissimilarity of the gut microbiota, beta-diversity measures were calculated, through the Bray-Curtis metric, which describes how many species are shared between samples. All Bray-Curtis dissimilarities were summarized by means of Kruskal’s non-metric MultiDimensional Scaling (MDS) method and were graphically represented into a bi-dimensional plot, where each point (defined by X and Y coordinates) represents an individual. Subjects who belonged to different groups (e.g., regions) were marked by different colors. To measure how similar an individual is to its own group (cohesion) compared to other groups (separation), the individual silhouette was estimated and the mean of all individual silhouettes within each group was computed. The individual silhouette ranges from −1 to +1, where a high value indicates that the individual is well matched to its own group and poorly matched to neighboring groups. If most individuals have a high silhouette value, then the clustering configuration is appropriate. On the contrary, if many individuals have a low or negative value, then the clustering configuration may have too many or too few groups.

A *p*-value < 0.05 was considered for statistical significance. Statistical analyses and plots were performed using the computing environment R (R Development Core Team, Vienna, Austria 2008, version 3.5.1.)

## 3. Results

### 3.1. Microbiota Profile in Italian Subjects from Different Regions

Demographic and behavioral characteristics of the study participants grouped by regions were reported in Table 1. 

**Figure 1:**
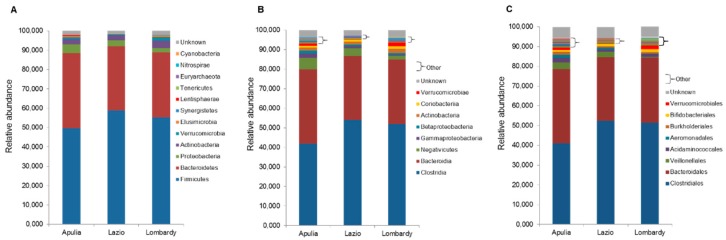
Microbiota composition of Italian healthy subjects grouped by regions, at the phylum (**A**), class (**B**) and order (**C**) levels. The mean value of all the detected taxa at each level is represented.

**Figure 2:**
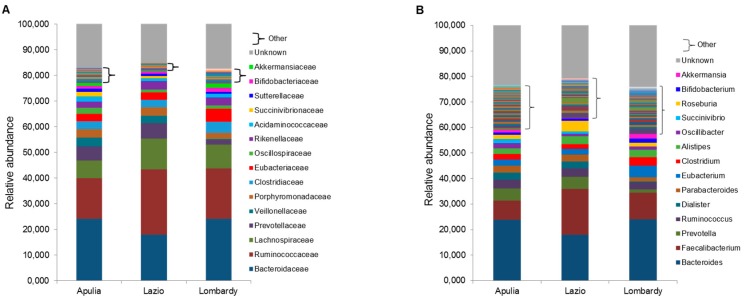
Microbiota composition of Italian healthy subjects grouped by regions, at the family (**A**) and genus (**B**) levels. The mean value of all the detected taxa at each level is represented.

The microbiota profile at all taxonomic levels was characterized for each of the 31 healthy individuals, and the mean relative abundance of bacterial taxa in Apulia, Lazio and Lombardy were represented in Figure 1 ((**A**)—phylum level, (**B**)—class level, (**C**)—order level) and in Figure 2 ((**A**)—family level, (**B**)—genus level; species level not shown)).

No significant differences were found among the three groups as for gender distribution, body mass index (BMI), type of diet, smoking habits, alcohol consumption and physical activity whereas a statistically significant difference was found with respect to participants’ age (*p* = 0.005), where subjects in Lazio were significantly younger than the subjects who belonged to the other regions (median: 37 vs. 26 vs. 42 years in Apulia, Lazio and Lombardy, respectively). When the participants were divided according to the three age classes, significant associations between relative abundances of microbial taxa and age classes were found for the families of *Acholeplasmataceae*, *Bacillaceae*, *Peptostreptococcaceae*, *Pseudomonadaceae,* for the genera of *Acetivibrio*, *Bacillus*, *Defluviitalea*, *Eggerthella*, *Fenollaria*, *Hydrogenoanaerobacterium*, *Lachnotalea*, *Lutispora*, *Natranaerovirga*, *Paludibacter*, *Porphyromonas*, *Pseudomonas*, *Raoultibacter* and for the species of *Alistipes finegoldii*, *Anaerotruncus rubiinfantis*, *Bacteroides acidifaciens*, *Bacteroides clarus*, *Bacteroides* sp. ANH 2438, *Bifidobacterium* sp. 113, *Blautia luti*, *Butyricimonas* sp. 180-3, *Butyrivibrio crossotus*, *Clostridium* sp. Culture Jar-19, *Dialister* sp. GBA27, *Eubacterium coprostanoligenes*, *Faecalibacterium prausnitzii*, *Fenollaria timonensis*, *Flintibacter butyricus*, *Prevotella* sp. 109, *Robinsoniella* sp. MCWD5, *Ruminococcus* sp. DJF_VR70k1 and *Ruminococcus* sp. ID1 (Table 2).

Furthermore, Spearman’s correlation coefficients were estimated to investigate the presence of a linear association between subjects’ age (considered as continuous variable) and the relative abundances of microorganisms. Indeed, the correlation analysis corroborates group comparisons analysis (with respect to age classes): while correlation achieves the highest statistical power in the detection of a linear trend, the group comparison analysis allow the detection of any potential non-linear associations. Statistically significant positive correlations with the age were found for *Acholeplasmataceae*, *Bacillaceae*, *Peptostreptococcaceae*, *Acetivibrio*, *Bacillus*, *Defluviitalea*, *Eggerthella*, *Lachnotalea*, *Natranaerovirga*, *Paludibacter*, *Raoultibacter*, *Bacteroides clarus*, *Bacteroides* sp. ANH. 2438, *Eubacterium coprostanoligenes*, *Flintibacter butyricus*, *Robinsoniella* sp. MCWD5 and *Ruminococcus* sp. ID, while a negative correlation was found for *Alistipes finegoldii*, *Bifidobacterium* sp. 113, *Blautia luti*, *Butyricimonas* sp. 180-3, *Butyrivibrio crossotus*, *Dialister* sp. GBA27 and *Ruminococcus* sp. DJF_VR70k1 (Table 2). These results strongly support that age influences gut microbiota composition. Conversely, no statistically significant difference in the microbial pattern was detected according to gender, alcohol consumption, BMI, type of diet (if Mediterranean or other), smoking habits and physical activity (data not shown). Once assessed that no other registered/collected demographic or behavioral characteristics of the subjects but age affected the fecal microbiota, we next analyzed its composition in relation to the geographical origin of the participants. The phyla of *Cyanobacteria* and *Nitrospirae*, the classes of *Epsilonproteobacteria*, *Nitrospira*, *Oligosphaeria* and *Sphingobacteriia* and the orders of *Alteromonadales*, *Anaeroplasmatales*, *Bacillales*, *Corynebacteriales*, *Desulfobacterales*, *Desulfurellales*, *Micrococcales*, *Myxococcales*, *Nautiliales*, *Nitrospirales*, *Sphingobacteriales*, *Streptomycetales*, *Synergistales*, *Syntrophobacterales*, *Thermoanaerobacterales*, *Tissierellales*, *Veillonellales* and *Vibrionales* resulted differently represented among the Apulia, Lazio and Lombardy groups (see Table 3).

**Figure 3:**
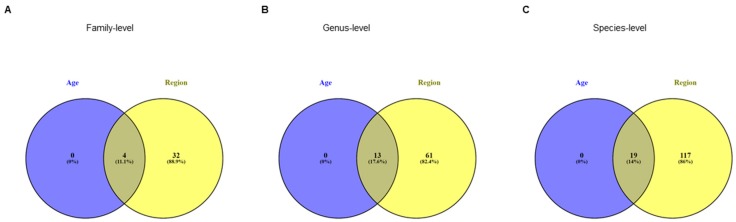
Venn diagrams showing the number of distinct and shared families (**A**), genera (**B**) and species (**C**) between subjects grouped by age classes and by regions.

Many differences emerged also at lower taxonomic levels, some of which completely overlapping with the ones found in the classification by age groups (Figure 3).

Having excluded possible spurious results (i.e., all those associations which were also significant with respect to age classes), *Aeromonadaceae, Anaeroplasmataceae, Atopobiaceae, Catabacteriaceae, Christensenellaceae, Clostridiales Family XII Incertae Sedis, Clostridiales Family XIII Incertae Sedis, Cytophagaceae, Desulfohalobiaceae, Desulfurellaceae, Enterococcaceae, Erwiniaceae, Flavobacteriaceae, Nautiliaceae, Nitrospiraceae, Oxalobacteraceae, Paenibacillaceae, Peptococcaceae, Polyangiaceae, Propionibacteriaceae, Puniceicoccaceae, Ruminococcaceae, Selenomonadaceae, Sphingobacteriaceae, Streptomycetaceae, Succinivibrionaceae, Synergistaceae, Syntrophorhabdaceae, Thermoanaerobacterales, Family III Incertae Sedis, Tissierellaceae, Veillonellaceae* and *Vibrionaceae* remained differently represented at the family level among the three regions. At the genus level, significant differences were found for *Acetanaerobacterium, Acetobacteroides, Aeromonas, Alkaliphilus, Alloprevotella, Anaerofilum, Anaeroplasma, Anaerostipes, Candidatus Phytoplasma, Candidatus Soleaferrea, Catabacter, Christensenella, Coprobacillus, Dakarella, Denitrobacterium, Desulfohalobium, Desulfotomaculum, Dialister, Dysgonomonas, Escherichia, Eubacterium, Faecalibacterium, Falcatimonas, Fastidiosipila, Gabonibacter, Gorbachella, Gordonibacter, Haemophilus, Harryflintia, Hespellia, Hippea, Howardella, Hungatella, Ihubacter, Intestinibacter, Lachnobacterium, Libanicoccus, Mediterranea, Megamonas, Megasphaera, Mitsuokella, Mucilaginibacter, Nautilia, Nitrospira, Olsenella, Oribacterium, Oxalobacter, Paenibacillus, Pantoea, Parasporobacterium, Peptococcus, Prevotellamassilia, Propionibacterium, Provencibacterium, Robinsoniella, Saccharofermentans, Selenomonas, Succinivibrio, Synergistes, Syntrophorhabdus* and *Wigglesworthia*. Finally, a total of 117 bacterial species were significantly different among the three regions (see Table 3, possible spurious results at the species level are underlined).

### 3.2. Bacterial Diversity

**Figure 4:**
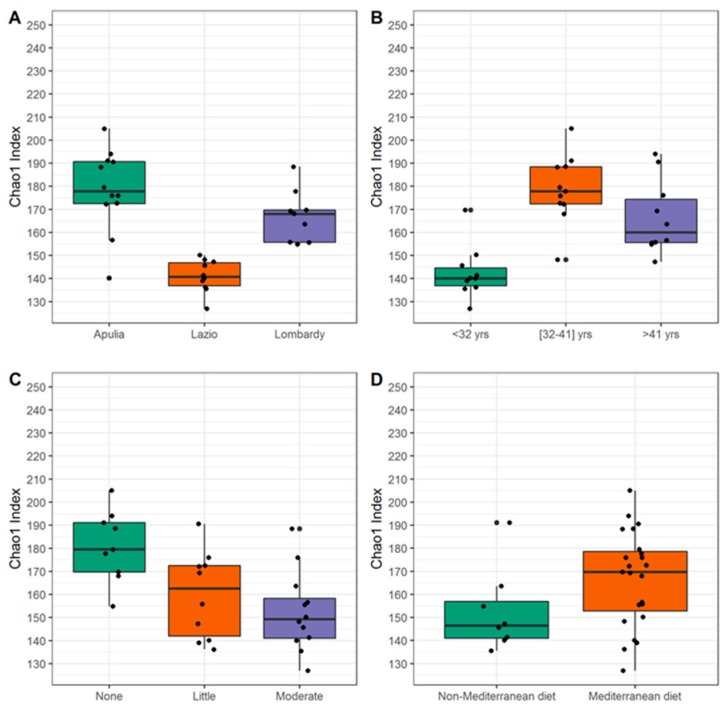
Box-plots of Chao1 index of species richness in Italian healthy subjects grouped by regions of origin (**A**), age classes (**B**), physical activity (**C**) and type of diet (**D**).

The species richness (i.e., Chao1 index) significantly differed when subjects were classified by their region of origin (median richness: 177.8 vs. 140.7 vs. 168.0 in Apulia, Lazio and Lombardy; *p* = 5.7 × 10^−5^), age classes (median richness: 140.1 vs. 177.8 vs. 160.0 in subjects < 32, 32–41 and > 41 years; *p* = 1.6 × 10^−4^) and physical activity (median richness: 179.5 vs. 162.5 vs. 149.2 in subjects who performed “none”, “little” and “moderate” physical activity; *p* = 6.8 × 10^−4^), whereas an interesting trend was observed when individuals were grouped according to their adherence to Mediterranean diet rather than to other non-Mediterranean styles (*p* = 0.07) (Figure 4).

**Figure 5:**
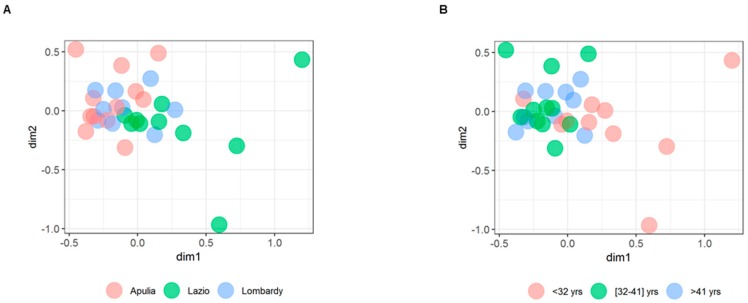
Kruskal’s non-metric MultiDimensional Scaling (MDS) plots of the Bray-Curtis dissimilarities in Italian healthy subjects grouped by regions of origin (**A**) and age classes (**B**). Each point plotted into the bi-dimensional space (as X, Y coordinates) represents an individual. Individuals that are more similar to one another are ordinated closer together and those who belong to different groups were marked by different colors. Mean silhouette value was 0.0069 for clustering by regions and −0.0225 for clustering by age classes.

Conversely, independently from the classification criterion, no statistically significant difference was found for the Shannon index, which takes into account the number and the relative abundance of the species within each sample (data not shown). Furthermore, as for beta-diversity, Kruskal’s non-metric MultiDimensional Scaling (MDS) plots of the Bray-Curtis dissimilarities did not reveal any significant clustering neither by regions (Figure 5A) and age classes (Figure 5B) nor by other classification criteria, as indicated by mean silhouette values around zero. The lack of Shannon index and beta diversity association may due to the sampling of multiple village sites and multiple cities within the study.

**Figure 6:**
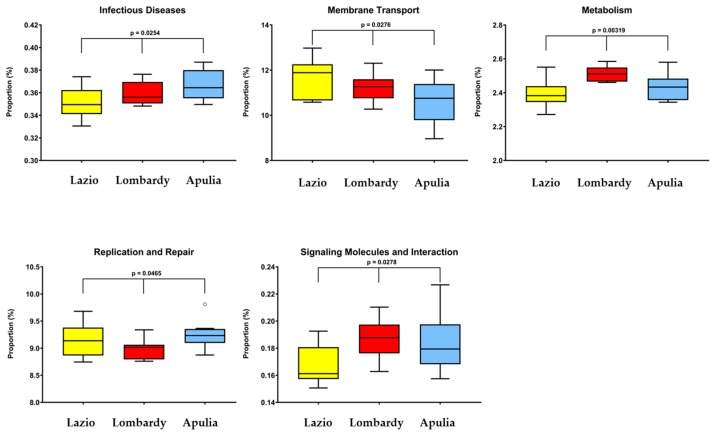
PICRUSt prediction of the functional composition of microbial communities grouped by region. A total of five KEGG pathways were significantly changed among the three regions.

In order to understand the functional meaning of the microbiota differences observed among the three regions, a PICRUSt prediction of the metagenomes was performed. A total of five pathways, namely infectious diseases, membrane transport, metabolism, replication and repair and signaling molecules and interactions emerged as significantly changed (Figure 6).

## 4. Discussion

It is well recognized that ethnicity and geographical locations are key factors influencing the composition and diversity of the gut microbiota [5]. However, beside understandable divergences across wide international geographical areas characterized by different socio-economic settings [16,17,18,19], important differences are emerging even in people with similar genetic and cultural background [21]. In this regard, the aim of the present study was to characterize the gut microbiota composition of healthy people belonging to three different Italian regions, namely Apulia, Lazio and Lombardy from the South, Center and North of the peninsula, respectively. All the study participants had the same ethnicity and were quite homogeneously distributed across the three regions as regards gender, BMI, physical activity, dietary, smoking and drinking habits. Only the age was not distributed similarly among the three regions, and since the age is an established factor influencing the composition of microbiota [12,19,29], it was taken into account as a potential confounding factor. However, it should be considered that, although gut microbiota composition changes throughout life, major shifts are described to occur in the transition from infancy to adulthood and then to old age, while it is documented to be quite stable through different stages of adult life [30,31,32]. Furthermore, differences emerged in the species richness of gut microbial communities according to the region, age class and physical activity criterion. Nevertheless, when we grouped our study population by age classes, differences in composition within the bacterial families, genera and species emerged. Although divided in three classes of age, our study population is mainly composed of young adults and adults (between 24 and 47 years of age with the exception of two individuals of 64 and 65 years old respectively), and includes only 1 subject older than 70 (which is considered the threshold age for defining an individual as elderly). We speculate that the significant differences observed in Chao1 index according to age are likely due to the acquisition of a mature (not aged) microbiota (Figure 4B).

Physical exercise is known to be associated to a healthier and more diverse microbiota, which seems to be in contrast with the results from our study. It should be considered, however, that in our study population people performing little and moderate physical activity overlapped with younger age, which would explain the reduced richness observed. All these and many other differences, also within higher taxonomic levels, were observed when the 31 subjects were grouped by regions. Noteworthy is that many taxa, which were represented in almost all the members of one or two regions, were instead completely absent in the other(s), supporting that gut microbiota and geographic localization are closely interlinked; this is, for example, the case of *Cyanobacteria* which were unique to Lombards, *Nitrospirae* only found in Apulians, and many other lower taxa. A predictive analysis of the functional pathways affected by the microbiota diversity among the three regions revealed five categories significantly changed. It should be kept in mind, however, that PICRUSt only performs predictions and that more accurate functional profiles require metagenomic approaches which will be the subject of future studies.

Our data, emphasize the importance of the selective pressure in shaping gut microbial ecology by numerous common environmental exposures. This could be the reflection of the different exposure of these individuals to industrial presence [33] (considering the north part more industrialized as compared to the south of Italy) agricultural chemicals like fertilizers (as the southern part of Italy is an agriculture-based Economy) or the natural source of water people drinks. As specified above, our study population was homogeneous from the ethnical point of view, so we can isolate the geographical effect from the ethnical factor. A very interesting point of view in trying to understand the bases of such a spatial microbiota variability comes from the application of the ecological theory according to which local diversification of microbiota could be introduced by processes of community ecology, such as dispersal, diversification, environmental selection and ecological drift, as exhaustively discussed by Costello et al. [34]. Further investigation would be required to shed light on the factors underlying such a difference in microbiota composition among the Italian regions.

## 5. Conclusions

Overall, our results point out the existence of a variability in the microbiota composition of populations closely related from geographical point of view. This interesting link between small-scale geography and gut microbiota deserves further investigation and poses important implications for the development of microbiota-based clinical approaches. Indeed, the role of microbiota in the onset, progression and response to therapies in a large number of diseases, is increasingly recognized both as a diagnostic marker [22,24,26] and as a manipulable target for improving the clinical course of pathologies [23,35]. Therefore, considering the existence of a variability within a limited geographic area could be particularly important in order to set up tailored therapeutic approaches. Moreover, attention should be paid when setting a specific microbial pattern as a reference for health or disease, since it may be strongly influenced by the population used to generate the data [21]. Further efforts should be devoted to identify the factors underlying the association between microbiota and small-scale geography [36].

## Figures and Tables

**Table 1:** Subjects’ demographic and behavioral characteristics by Italian regions.

		Apulia (N = 12)	Lazio (N = 10)	Lombardy (N = 9)	*p*-Value
Age (years)	Median [IQR]	37.0 [35.0–44.2]	26.0 [24.0–29.0]	42.0 [39.0–47.0]	0.005 *
	Range	28–65	24–46	29–74	
Gender—N (%)	Males	6 (50.0)	6 (60.0)	3 (33.3)	0.542 ^#^
BMI (Kg/m^2^)	Mean ± SD	22.8 ± 2.3	22.3 ± 3.0	21.7 ± 3.5	0.723 ^$^
	Range	19.1–28.1	17.2–26.9	16.5–28.4	
BMI (WHO classification)—N (%)	Underweight (<18.5)	0 (0.0)	2 (20.0)	2 (22.2)	0.528 ^#^
	Normal weight (18.5–24.9)	10 (83.3)	7 (70.0)	6 (66.7)	
	Overweight (25.0–29.9)	2 (16.7)	1 (10.0)	1 (11.1)	
Diet (%)	Mediterranean	11 (91.7)	5 (50.0)	7 (77.8)	0.149 ^#^
	Hypercaloric	1 (8.3)	0 (0.0)	1 (11.1)	
	Hypocaloric	0 (0.0)	1 (10.0)	1 (11.1)	
	Vegetarian	0 (0.0)	1 (10.0)	0 (0.0)	
	Iperproteic (meat)	0 (0.0)	1 (10.0)	0 (0.0)	
	Carbohydrates and proteins, little vegetables	0 (0.0)	2 (20.0)	0 (0.0)	
Smoking (n.cigarettes/day)	Median [IQR]	0.0 [0.0–0.8]	0.0 [0.0–1.5]	0.0 [0.0–11.0]	0.764 *
	Range	0–12	0–5	0–17	
Smokers (yes vs. no)—N (%)	Yes	3 (25.0)	3 (30.0)	3 (33.3)	1.000 ^#^
Alcohol—N (%)	None	3 (25.0)	4 (40.0)	1 (11.1)	0.336 ^#^
	Little (1 occasional glass)	9 (75.0)	5 (50.0)	6 (66.7)	
	Moderate (1–2 glasses/day)	0 (0.0)	1 (10.0)	2 (22.2)	
Physical activity—N (%)	None	4 (33.3)	0 (0.0)	5 (55.6)	0.051 ^#^
	Little	5 (41.7)	3 (30.0)	2 (22.2)	
	Moderate	3 (25.0)	7 (70.0)	2 (22.2)	

SD: Standard deviation; IQR: Interquartile range (i.e., first-third quartiles); * *p*-value from Kruskal-Wallis test; $ *p*-value from ANOVA model; # *p*-value from Fisher exact test.

**Table 2:** Gut microbiota in subjects grouped by age classes (i.e., tertiles) and Spearman correlation coefficients. Data were reported as median along with interquartile range (first-third quartiles). Only significant results (i.e., *p*-values < 0.05 from any statistical test) were reported. All *p*-values were adjusted controlling for the False Discovery Rate at 0.05 level within each taxonomic level.

						Spearman Correlation	
Biological Classification	Microbiota	Age < 32 Years (N = 10)	Age 32–41 Years (N = 11)	Age > 41 Years (N = 10)	Test for Overall Difference (*p*-Value ^#^)	Coefficient	*p*-Value
Family	Acholeplasmataceae	0.0000 [0.0000, 0.0000]	0.0027 [0.0006, 0.0155]	0.0073 [0.0035, 0.0514]	0.025	0.604	0.021
	Bacillaceae	0.0000 [0.0000, 0.0000]	0.0184 [0.0094, 0.0219]	0.0188 [0.0071, 0.0295]	0.025	0.566	0.029
	Peptostreptococcaceae	0.0000 [0.0000, 0.0000]	0.0274 [0.0193, 0.0426]	0.0364 [0.0222, 0.0432]	0.041	0.536	0.041
	Pseudomonadaceae	0.0000 [0.0000, 0.0000]	0.0168 [0.0090, 0.0185]	0.0129 [0.0095, 0.0263]	0.025	0.457	0.090
Genus	*Acetivibrio*	0.0000 [0.0000, 0.0000]	0.0142 [0.0086, 0.0238]	0.0128 [0.0075, 0.0222]	0.028	0.551	0.041
	*Bacillus*	0.0000 [0.0000, 0.0000]	0.0092 [0.0045, 0.0144]	0.0059 [0.0032, 0.0170]	0.028	0.543	0.041
	*Defluviitalea*	0.0000 [0.0000, 0.0000]	0.0114 [0.0032, 0.0197]	0.0225 [0.0064, 0.0257]	0.028	0.649	0.012
	*Eggerthella*	0.0000 [0.0000, 0.0000]	0.0083 [0.0019, 0.0379]	0.0112 [0.0066, 0.0182]	0.056	0.524	0.048
	*Fenollaria*	0.0000 [0.0000, 0.0000]	0.0176 [0.0131, 0.0201]	0.0085 [0.0060, 0.0145]	0.039	0.471	0.089
	*Hydrogenoanaerobacterium*	0.0000 [0.0000, 0.0000]	0.0193 [0.0070, 0.0257]	0.0099 [0.0062, 0.0163]	0.034	0.426	0.131
	*Lachnotalea*	0.0000 [0.0000, 0.0000]	0.0088 [0.0063, 0.0143]	0.0087 [0.0047, 0.0269]	0.028	0.580	0.041
	*Lutispora*	0.0000 [0.0000, 0.0000]	0.0054 [0.0030, 0.0186]	0.0068 [0.0020, 0.0246]	0.034	0.499	0.067
	*Natranaerovirga*	0.0000 [0.0000, 0.0000]	0.0183 [0.0110, 0.0311]	0.0200 [0.0123, 0.0215]	0.028	0.528	0.048
	*Paludibacter*	0.0000 [0.0000, 0.0000]	0.0048 [0.0024, 0.0078]	0.0045 [0.0025, 0.0060]	0.028	0.558	0.041
	*Porphyromonas*	0.0000 [0.0000, 0.0000]	0.0251 [0.0156, 0.0273]	0.0114 [0.0071, 0.0239]	0.040	0.418	0.136
	*Pseudomonas*	0.0000 [0.0000, 0.0000]	0.0143 [0.0079, 0.0171]	0.0119 [0.0094, 0.0215]	0.033	0.482	0.085
	*Raoultibacter*	0.0000 [0.0000, 0.0000]	0.0043 [0.0011, 0.0139]	0.0113 [0.0045, 0.0212]	0.050	0.548	0.041
Species	*Alistipes finegoldii*	0.1246 [0.0709, 0.6407]	0.0038 [0.0010, 0.0653]	0.0022 [0.0001, 0.0313]	0.049	−0.559	0.043
	*Anaerotruncus rubiinfantis*	0.0000 [0.0000, 0.0000]	0.0081 [0.0034, 0.0272]	0.0076 [0.0034, 0.0120]	0.049	0.466	0.091
	*Bacteroides acidifaciens*	0.0000 [0.0000, 0.0000]	0.0193 [0.0138, 0.0321]	0.0242 [0.0162, 0.0305]	0.049	0.521	0.056
	*Bacteroides clarus*	0.0000 [0.0000, 0.0000]	0.0035 [0.0028, 0.0559]	0.0066 [0.0015, 0.0176]	0.049	0.551	0.043
	*Bacteroides* sp. ANH. 2438	0.0000 [0.0000, 0.0000]	0.0005 [0.0000, 0.0012]	0.0010 [0.0000, 0.0161]	0.189	0.537	0.043
	*Bifidobacterium* sp. 113	0.0012 [0.0001, 0.0197]	0.0000 [0.0000, 0.0000]	0.0000 [0.0000, 0.0000]	0.048	−0.580	0.043
	*Blautia luti*	0.0032 [0.0003, 0.1185]	0.0000 [0.0000, 0.0000]	0.0000 [0.0000, 0.0000]	0.035	−0.637	0.012
	*Butyricimonas* sp. 180 3	0.0013 [0.0000, 0.0079]	0.0000 [0.0000, 0.0000]	0.0000 [0.0000, 0.0000]	0.072	−0.538	0.043
	*Butyrivibrio crossotus*	0.0017 [0.0007, 0.0027]	0.0000 [0.0000, 0.0000]	0.0000 [0.0000, 0.0000]	0.048	−0.554	0.043
	*Clostridium* sp. Culture Jar 19	0.0000 [0.0000, 0.0000]	0.0177 [0.0053, 0.0322]	0.0066 [0.0036, 0.0087]	0.048	0.447	0.097
	*Dialister* sp. GBA27	0.0008 [0.0000, 0.0090]	0.0000 [0.0000, 0.0000]	0.0000 [0.0000, 0.0000]	0.049	−0.536	0.043
	*Eubacterium coprostanoligenes*	0.0000 [0.0000, 0.0000]	0.0326 [0.0159, 0.0655]	0.0817 [0.0316, 0.3701]	0.058	0.573	0.043
	*Faecalibacterium prausnitzii*	12.7134 [10.7228, 14.3182]	3.3569 [2.0414, 5.4147]	5.3014 [3.4161, 6.9285]	0.035	−0.517	0.057
	*Fenollaria timonensis*	0.0000 [0.0000, 0.0000]	0.0154 [0.0118, 0.0183]	0.0074 [0.0053, 0.0129]	0.049	0.477	0.082
	*Flintibacter butyricus*	0.0000 [0.0000, 0.0000]	0.0099 [0.0011, 0.0127]	0.0184 [0.0145, 0.0311]	0.035	0.664	0.008
	*Prevotella* sp. 109	0.0000 [0.0000, 0.0000]	0.0025 [0.0002, 0.0068]	0.0055 [0.0012, 0.2678]	0.048	0.503	0.064
	*Robinsoniella* sp. MCWD5	0.0000 [0.0000, 0.0000]	0.0143 [0.0044, 0.0239]	0.0156 [0.0081, 0.0296]	0.049	0.564	0.043
	*Ruminococcus* sp. DJF_VR70k1	0.0011 [0.0002, 0.0087]	0.0000 [0.0000, 0.0000]	0.0000 [0.0000, 0.0000]	0.048	−0.537	0.043
	*Ruminococcus* sp. ID1	0.0000 [0.0000, 0.0000]	0.0221 [0.0109, 0.0261]	0.0292 [0.0172, 0.0674]	0.048	0.659	0.008

^#^ KW: *p*-value from Kruskal-Wallis test.

**Table 3:** Gut microbiota in subjects grouped by Italian regions. Data were reported as median along with interquartile range (first-third quartiles). Only significant results (i.e., *p*-values < 0.05 from any statistical test) were reported. All p-values were adjusted controlling for the False Discovery Rate at 0.05 level within each taxonomic level.

Biological Classification	Microbiota	Apulia (N = 12)	Lazio (N = 10)	Lombardy (N = 9)	Test for Overall Difference (*p*-Value *)
Phylum	Cyanobacteria	0.0000 [0.0000, 0.0000]	0.0000 [0.0000, 0.0000]	0.0031 [0.0011, 0.0064]	<0.001
	Nitrospirae	0.0037 [0.0019, 0.0061]	0.0000 [0.0000, 0.0000]	0.0000 [0.0000, 0.0000]	<0.001
Class	Epsilonproteobacteria	0.0241 [0.0070, 0.0465]	0.0000 [0.0000, 0.0000]	0.0086 [0.0080, 0.0258]	<0.001
	Nitrospira	0.0036 [0.0019, 0.0061]	0.0000 [0.0000, 0.0000]	0.0000 [0.0000, 0.0000]	<0.001
	Oligosphaeria	0.0007 [0.0000, 0.0152]	0.0000 [0.0000, 0.0000]	0.0000 [0.0000, 0.0000]	0.024
	Sphingobacteriia	0.0127 [0.0100, 0.0253]	0.0000 [0.0000, 0.0000]	0.0078 [0.0074, 0.0156]	<0.001
Order	Alteromonadales	0.0132 [0.0091, 0.0170]	0.0000 [0.0000, 0.0000]	0.0125 [0.0108, 0.0188]	<0.001
	Anaeroplasmatales	0.0000 [0.0000, 0.0000]	0.0000 [0.0000, 0.0000]	0.0009 [0.0000, 0.0183]	0.003
	Bacillales	0.0911 [0.0763, 0.1214]	0.0255 [0.0195, 0.0356]	0.0464 [0.0263, 0.0607]	0.006
	Corynebacteriales	0.0062 [0.0046, 0.0112]	0.0000 [0.0000, 0.0000]	0.0086 [0.0063, 0.0125]	<0.001
	Desulfobacterales	0.0000 [0.0000, 0.0000]	0.0000 [0.0000, 0.0000]	0.0064 [0.0009, 0.0260]	<0.001
	Desulfurellales	0.0023 [0.0004, 0.0130]	0.0000 [0.0000, 0.0000]	0.0000 [0.0000, 0.0000]	<0.001
	Micrococcales	0.0180 [0.0133, 0.0248]	0.0000 [0.0000, 0.0000]	0.0151 [0.0108, 0.0184]	<0.001
	Myxococcales	0.0008 [0.0000, 0.0083]	0.0000 [0.0000, 0.0000]	0.0000 [0.0000, 0.0000]	0.001
	Nautiliales	0.017 [0.0035, 0.0287]	0.0000 [0.0000, 0.0000]	0.0048 [0.0018, 0.0168]	0.001
	Nitrospirales	0.0033 [0.0017, 0.0060]	0.0000 [0.0000, 0.0000]	0.0000 [0.0000, 0.0000]	<0.001
	Sphingobacteriales	0.0103 [0.0082, 0.0230]	0.0000 [0.0000, 0.0000]	0.0074 [0.0060, 0.0156]	<0.001
	Streptomycetales	0.0247 [0.0167, 0.0394]	0.0080 [0.0051, 0.0097]	0.0311 [0.0091, 0.0478]	0.044
	Synergistales	0.0095 [0.0054, 0.0969]	0.0000 [0.0000, 0.0000]	0.0106 [0.0032, 0.0201]	<0.001
	Syntrophobacterales	0.0007 [0.0000, 0.0097]	0.0000 [0.0000, 0.0000]	0.0000 [0.0000, 0.0000]	0.001
	Thermoanaerobacterales	0.0060 [0.0028, 0.0126]	0.0000 [0.0000, 0.0000]	0.0041 [0.0028, 0.0314]	<0.001
	Tissierellales	0.0137 [0.0118, 0.0175]	0.0000 [0.0000, 0.0000]	0.0124 [0.0070, 0.0401]	<0.001
	Veillonellales	2.9426 [0.2835, 4.7890]	1.8227 [0.1197, 5.6735]	0.0389 [0.0156, 0.1560]	0.033
	Vibrionales	0.0026 [0.0020, 0.0073]	0.0000 [0.0000, 0.0000]	0.0000 [0.0000, 0.0000]	<0.001
Family	Acholeplasmataceae	0.0049 [0.0010, 0.0149]	0.0000 [0.0000, 0.0000]	0.0052 [0.0032, 0.1064]	<0.001
	Aeromonadaceae	0.0000 [0.0000, 0.0000]	0.0000 [0.0000, 0.0000]	0.0134 [0.0048, 0.0156]	<0.001
	Anaeroplasmataceae	0.0000 [0.0000, 0.0000]	0.0000 [0.0000, 0.0000]	0.0009 [0.0000, 0.0118]	0.002
	Atopobiaceae	0.0045 [0.0016, 0.0115]	0.0029 [0.0006, 0.0207]	0.0000 [0.0000, 0.0000]	0.002
	Bacillaceae	0.0171 [0.0072, 0.0244]	0.0000 [0.0000, 0.0000]	0.0186 [0.0069, 0.0220]	<0.001
	Catabacteriaceae	0.0327 [0.0234, 0.0440]	0.0081 [0.0035, 0.0175]	0.0126 [0.0037, 0.0343]	0.035
	Christensenellaceae	0.6797 [0.2907, 1.3801]	0.1033 [0.0223, 0.2216]	0.2123 [0.0957, 0.3820]	0.035
	Clostridiales Family XII. Incertae Sedis	0.0063 [0.0013, 0.0232]	0.0000 [0.0000, 0.0000]	0.0000 [0.0000, 0.0000]	<0.001
	Clostridiales Family XIII. Incertae Sedis	0.0000 [0.0000, 0.0000]	0.0206 [0.0125, 0.0302]	0.0092 [0.0060, 0.0419]	<0.001
	Cytophagaceae	0.0000 [0.0000, 0.0000]	0.0000 [0.0000, 0.0000]	0.0028 [0.0023, 0.0371]	<0.001
	Desulfohalobiaceae	0.0036 [0.0028, 0.0167]	0.0000 [0.0000, 0.0000]	0.0000 [0.0000, 0.0000]	<0.001
	Desulfurellaceae	0.0023 [0.0004, 0.0127]	0.0000 [0.0000, 0.0000]	0.0000 [0.0000, 0.0000]	<0.001
	Enterococcaceae	0.0047 [0.0010, 0.0218]	0.0000 [0.0000, 0.0000]	0.0060 [0.0016, 0.0113]	0.001
	Erwiniaceae	0.0097 [0.0082, 0.0216]	0.0000 [0.0000, 0.0000]	0.0142 [0.0128, 0.0239]	<0.001
	Flavobacteriaceae	0.0107 [0.0070, 0.0145]	0.0000 [0.0000, 0.0000]	0.0093 [0.0050, 0.0150]	<0.001
	Nautiliaceae	0.0177 [0.0035, 0.0277]	0.0000 [0.0000, 0.0000]	0.0044 [0.0018, 0.0161]	0.001
	Nitrospiraceae	0.0029 [0.0017, 0.0058]	0.0000 [0.0000, 0.0000]	0.0000 [0.0000, 0.0000]	<0.001
	Oxalobacteraceae	0.0020 [0.0000, 0.0125]	0.0000 [0.0000, 0.0000]	0.0226 [0.0016, 0.0436]	0.002
	Paenibacillaceae	0.0161 [0.0085, 0.0247]	0.0000 [0.0000, 0.0000]	0.0000 [0.0000, 0.0000]	<0.001
	Peptococcaceae	0.0379 [0.0230, 0.0551]	0.0035 [0.0026, 0.0224]	0.0074 [0.0055, 0.0103]	0.007
	Peptostreptococcaceae	0.0331 [0.0256, 0.0396]	0.0000 [0.0000, 0.0000]	0.0418 [0.0194, 0.0707]	<0.001
	Polyangiaceae	0.0002 [0.0000, 0.0052]	0.0000 [0.0000, 0.0000]	0.0000 [0.0000, 0.0000]	0.009
	Propionibacteriaceae	0.1445 [0.0063, 0.2139]	0.0026 [0.0014, 0.0043]	0.2381 [0.0218, 0.6672]	0.023
	Pseudomonadaceae	0.0168 [0.0100, 0.0303]	0.0000 [0.0000, 0.0000]	0.0140 [0.0100, 0.0177]	<0.001
	Puniceicoccaceae	0.0000 [0.0000, 0.0045]	0.0000 [0.0000, 0.0000]	0.0000 [0.0000, 0.0000]	0.023
	Ruminococcaceae	15.3334 [9.8807, 21.7498]	25.0671 [23.1509, 29.6044]	19.1784 [11.2163, 24.2467]	0.037
	Selenomonadaceae	0.0512 [0.0298, 0.1030]	0.0070 [0.0052, 0.0103]	0.0043 [0.0026, 0.0086]	0.023
	Sphingobacteriaceae	0.0088 [0.0071, 0.0220]	0.0000 [0.0000, 0.0000]	0.0000 [0.0000, 0.0000]	<0.001
	Streptomycetaceae	0.0206 [0.0123, 0.0365]	0.0078 [0.0042, 0.0096]	0.0280 [0.0091, 0.0473]	0.046
	Succinivibrionaceae	0.0010 [0.0000, 0.0019]	0.0007 [0.0006, 0.0010]	0.0000 [0.0000, 0.0000]	0.009
	Synergistaceae	0.0067 [0.0036, 0.0856]	0.0000 [0.0000, 0.0000]	0.0097 [0.0026, 0.0183]	<0.001
	Syntrophorhabdaceae	0.0003 [0.0000, 0.0085]	0.0000 [0.0000, 0.0000]	0.0000 [0.0000, 0.0000]	0.009
	Thermoanaerobacterales Family III Incertae Sedis	0.0008 [0.0000, 0.0014]	0.0000 [0.0000, 0.0000]	0.0000 [0.0000, 0.0000]	0.001
	Tissierellaceae	0.0000 [0.0000, 0.0000]	0.0000 [0.0000, 0.0000]	0.0060 [0.0029, 0.0097]	<0.001
	Veillonellaceae	2.9400 [0.2817, 4.7843]	1.8205 [0.1195, 5.6608]	0.0389 [0.0156, 0.1558]	0.025
	Vibrionaceae	0.0026 [0.0020, 0.0073]	0.0000 [0.0000, 0.0000]	0.0000 [0.0000, 0.0000]	<0.001
Genus	*Acetanaerobacterium*	0.0192 [0.0064, 0.0330]	0.0000 [0.0000, 0.0000]	0.0000 [0.0000, 0.0000]	<0.001
	*Acetivibrio*	0.0144 [0.0117, 0.0210]	0.0000 [0.0000, 0.0000]	0.0112 [0.0075, 0.0275]	<0.001
	*Acetobacteroides*	0.0002 [0.0000, 0.0032]	0.0000 [0.0000, 0.0000]	0.0000 [0.0000, 0.0000]	0.009
	*Aeromonas*	0.0000 [0.0000, 0.0000]	0.0000 [0.0000, 0.0000]	0.0107 [0.0032, 0.0149]	<0.001
	*Alkaliphilus*	0.0000 [0.0000, 0.0000]	0.0000 [0.0000, 0.0000]	0.0086 [0.0057, 0.0126]	<0.001
	*Alloprevotella*	0.0000 [0.0000, 0.0000]	0.0007 [0.0003, 0.0014]	0.0000 [0.0000, 0.0000]	<0.001
	*Anaerofilum*	0.0068 [0.0044, 0.0207]	0.0086 [0.0044, 0.0173]	0.0000 [0.0000, 0.0000]	<0.001
	*Anaeroplasma*	0.0000 [0.0000, 0.0000]	0.0000 [0.0000, 0.0000]	0.0000 [0.0000, 0.0091]	0.012
	*Anaerostipes*	0.0234 [0.0089, 0.0611]	0.0174 [0.0121, 0.0206]	0.1156 [0.0316, 0.5372]	0.024
	*Bacillus*	0.0097 [0.0049, 0.0155]	0.0000 [0.0000, 0.0000]	0.0065 [0.0031, 0.0124]	<0.001
	*Candidatus Phytoplasma*	0.0000 [0.0000, 0.0000]	0.0000 [0.0000, 0.0000]	0.0021 [0.0016, 0.0113]	<0.001
	*Candidatus Soleaferrea*	0.0122 [0.0083, 0.0572]	0.0091 [0.0075, 0.0119]	0.0000 [0.0000, 0.0000]	0.001
	*Catabacter*	0.0289 [0.0200, 0.0339]	0.0075 [0.0030, 0.0153]	0.0103 [0.0036, 0.0280]	0.036
	*Christensenella*	0.4438 [0.1379, 0.7502]	0.0479 [0.0142, 0.1074]	0.1088 [0.0463, 0.1956]	0.017
	*Coprobacillus*	0.0000 [0.0000, 0.0047]	0.0000 [0.0000, 0.0000]	0.0000 [0.0000, 0.0000]	0.024
	*Dakarella*	0.1114 [0.0015, 0.7843]	0.0002 [0.0000, 0.0004]	0.0000 [0.0000, 0.0000]	0.002
	*Defluviitalea*	0.0151 [0.0062, 0.0247]	0.0000 [0.0000, 0.0000]	0.0191 [0.0062, 0.0218]	<0.001
	*Denitrobacterium*	0.0000 [0.0000, 0.0000]	0.0036 [0.0000, 0.0198]	0.0020 [0.0005, 0.0075]	0.005
	*Desulfohalobium*	0.0009 [0.0003, 0.0049]	0.0000 [0.0000, 0.0000]	0.0000 [0.0000, 0.0000]	<0.001
	*Desulfotomaculum*	0.0077 [0.0036, 0.0154]	0.0000 [0.0000, 0.0000]	0.0000 [0.0000, 0.0000]	<0.001
	*Dialister*	1.7288 [0.0421, 4.6387]	1.7564 [0.0939, 5.6073]	0.0041 [0.0027, 0.0080]	0.016
	*Dysgonomonas*	0.0106 [0.0040, 0.0272]	0.0000 [0.0000, 0.0000]	0.0000 [0.0000, 0.0000]	<0.001
	*Eggerthella*	0.0069 [0.0026, 0.0144]	0.0000 [0.0000, 0.0000]	0.0195 [0.0145, 0.0349]	<0.001
	*Escherichia*	0.0029 [0.0009, 0.0262]	0.0000 [0.0000, 0.0000]	0.0011 [0.0000, 0.0089]	0.001
	*Eubacterium*	1.8114 [1.2982, 3.1914]	1.0085 [0.7616, 3.2710]	4.5088 [2.7865, 6.5613]	0.046
	*Faecalibacterium*	6.7009 [5.1594, 8.8515]	18.9963 [17.4550, 19.9556]	9.0591 [6.4928, 10.7255]	0.003
	*Falcatimonas*	0.0000 [0.0000, 0.0000]	0.0037 [0.0022, 0.0086]	0.0097 [0.0009, 0.0456]	<0.001
	*Fastidiosipila*	0.0041 [0.0017, 0.0068]	0.0000 [0.0000, 0.0000]	0.0011 [0.0002, 0.0022]	0.001
	*Fenollaria*	0.0136 [0.0057, 0.0178]	0.0000 [0.0000, 0.0000]	0.0203 [0.0124, 0.0289]	<0.001
	*Gabonibacter*	0.0000 [0.0000, 0.0000]	0.0003 [0.0000, 0.0009]	0.0000 [0.0000, 0.0000]	0.007
	*Gorbachella*	0.0000 [0.0000, 0.0000]	0.0000 [0.0000, 0.0000]	0.0172 [0.0093, 0.0202]	<0.001
	*Gordonibacter*	0.0000 [0.0000, 0.0000]	0.0000 [0.0000, 0.0000]	0.0186 [0.0021, 0.0473]	<0.001
	*Haemophilus*	0.0014 [0.0004, 0.0213]	0.0000 [0.0000, 0.0000]	0.0093 [0.0028, 0.0252]	0.001
	*Harryflintia*	0.0005 [0.0000, 0.0022]	0.0006 [0.0004, 0.0094]	0.0000 [0.0000, 0.0000]	0.005
	*Hespellia*	0.0075 [0.0027, 0.0117]	0.0161 [0.0109, 0.0229]	0.0000 [0.0000, 0.0000]	<0.001
	*Hippea*	0.0023 [0.0000, 0.0118]	0.0000 [0.0000, 0.0000]	0.0000 [0.0000, 0.0000]	0.001
	*Howardella*	0.0005 [0.0003, 0.0113]	0.0000 [0.0000, 0.0000]	0.0000 [0.0000, 0.0000]	<0.001
	*Hungatella*	0.0000 [0.0000, 0.0000]	0.0131 [0.0025, 0.0207]	0.0000 [0.0000, 0.0000]	<0.001
	*Hydrogenoanaerobacterium*	0.0177 [0.0089, 0.0243]	0.0000 [0.0000, 0.0000]	0.0158 [0.0052, 0.0193]	<0.001
	*Ihubacter*	0.0000 [0.0000, 0.0000]	0.0096 [0.0066, 0.0205]	0.0057 [0.0025, 0.0247]	<0.001
	*Intestinibacter*	0.0000 [0.0000, 0.0000]	0.0000 [0.0000, 0.0000]	0.0037 [0.0005, 0.0382]	<0.001
	*Lachnobacterium*	0.0000 [0.0000, 0.0000]	0.0118 [0.0080, 0.0242]	0.0187 [0.0104, 0.0365]	<0.001
	*Lachnotalea*	0.0084 [0.0070, 0.0119]	0.0000 [0.0000, 0.0000]	0.0156 [0.0050, 0.0294]	<0.001
	*Libanicoccus*	0.0000 [0.0000, 0.0000]	0.0000 [0.0000, 0.0004]	0.0000 [0.0000, 0.0000]	0.023
	*Lutispora*	0.0051 [0.0036, 0.0229]	0.0000 [0.0000, 0.0000]	0.0054 [0.0028, 0.0214]	0.001
	*Mediterranea*	0.0012 [0.0003, 0.0024]	0.0000 [0.0000, 0.0000]	0.0000 [0.0000, 0.0000]	<0.001
	*Megamonas*	0.0010 [0.0000, 0.0042]	0.0000 [0.0000, 0.0000]	0.0000 [0.0000, 0.0000]	0.001
	*Megasphaera*	0.0343 [0.0003, 0.5532]	0.0000 [0.0000, 0.0000]	0.0000 [0.0000, 0.0000]	<0.001
	*Mitsuokella*	0.0010 [0.0000, 0.0094]	0.0000 [0.0000, 0.0000]	0.0000 [0.0000, 0.0000]	0.001
	*Mucilaginibacter*	0.0000 [0.0000, 0.0019]	0.0000 [0.0000, 0.0000]	0.0000 [0.0000, 0.0000]	0.024
	*Natranaerovirga*	0.0177 [0.0100, 0.0249]	0.0000 [0.0000, 0.0000]	0.0208 [0.0183, 0.0279]	<0.001
	*Nautilia*	0.0129 [0.0029, 0.0231]	0.0000 [0.0000, 0.0000]	0.0034 [0.0014, 0.0054]	0.001
	*Nitrospira*	0.0013 [0.0000, 0.0029]	0.0000 [0.0000, 0.0000]	0.0000 [0.0000, 0.0000]	0.001
	*Olsenella*	0.0033 [0.0000, 0.0109]	0.0009 [0.0000, 0.0203]	0.0000 [0.0000, 0.0000]	0.024
	*Oribacterium*	0.0069 [0.0037, 0.0124]	0.0000 [0.0000, 0.0000]	0.0062 [0.0032, 0.0073]	<0.001
	*Oxalobacter*	0.0000 [0.0000, 0.0000]	0.0000 [0.0000, 0.0000]	0.0204 [0.0005, 0.0343]	<0.001
	*Paenibacillus*	0.0143 [0.0068, 0.0230]	0.0000 [0.0000, 0.0000]	0.0000 [0.0000, 0.0000]	<0.001
	*Paludibacter*	0.0054 [0.0025, 0.0116]	0.0000 [0.0000, 0.0000]	0.0046 [0.0023, 0.0048]	<0.001
	*Pantoea*	0.0016 [0.0007, 0.0103]	0.0000 [0.0000, 0.0000]	0.0000 [0.0000, 0.0000]	<0.001
	*Parasporobacterium*	0.0000 [0.0000, 0.0000]	0.0023 [0.0000, 0.0075]	0.0000 [0.0000, 0.0000]	0.002
	*Peptococcus*	0.0093 [0.0034, 0.0207]	0.0000 [0.0000, 0.0000]	0.0000 [0.0000, 0.0000]	<0.001
	*Porphyromonas*	0.0246 [0.0185, 0.0293]	0.0000 [0.0000, 0.0000]	0.0138 [0.0112, 0.0254]	<0.001
	*Prevotellamassilia*	0.0003 [0.0000, 0.0016]	0.0000 [0.0000, 0.0000]	0.0000 [0.0000, 0.0000]	0.009
	*Propionibacterium*	0.1439 [0.0041, 0.2118]	0.0000 [0.0000, 0.0000]	0.1904 [0.0156, 0.6649]	<0.001
	*Provencibacterium*	0.0000 [0.0000, 0.0006]	0.0000 [0.0000, 0.0000]	0.0006 [0.0000, 0.0011]	0.030
	*Pseudomonas*	0.0156 [0.0094, 0.0245]	0.0000 [0.0000, 0.0000]	0.0118 [0.0092, 0.0138]	<0.001
	*Raoultibacter*	0.0052 [0.0016, 0.0131]	0.0000 [0.0000, 0.0000]	0.0161 [0.0097, 0.0218]	<0.001
	*Robinsoniella*	0.0179 [0.0128, 0.0248]	0.0122 [0.0065, 0.0148]	0.0481 [0.0374, 0.1007]	0.016
	*Saccharofermentans*	0.0380 [0.0251, 0.0634]	0.0000 [0.0000, 0.0000]	0.0121 [0.0065, 0.0403]	<0.001
	*Selenomonas*	0.0061 [0.0027, 0.0218]	0.0000 [0.0000, 0.0000]	0.0000 [0.0000, 0.0000]	<0.001
	*Succinivibrio*	0.0008 [0.0000, 0.0017]	0.0007 [0.0006, 0.0010]	0.0000 [0.0000, 0.0000]	0.009
	*Synergistes*	0.0000 [0.0000, 0.0147]	0.0000 [0.0000, 0.0000]	0.0000 [0.0000, 0.0000]	0.024
	*Syntrophorhabdus*	0.0000 [0.0000, 0.0048]	0.0000 [0.0000, 0.0000]	0.0000 [0.0000, 0.0000]	0.024
	*Wigglesworthia*	0.0069 [0.0043, 0.0088]	0.0000 [0.0000, 0.0000]	0.0000 [0.0000, 0.0000]	<0.001
Species	*Clostridium colinum*	0.0000 [0.0000, 0.0054]	0.0000 [0.0000, 0.0000]	0.0000 [0.0000, 0.0000]	0.026
	*Clostridium glycyrrhizinilyticum*	0.0000 [0.0000, 0.0028]	0.0000 [0.0000, 0.0000]	0.0000 [0.0000, 0.0000]	0.026
	*Clostridium polysaccharolyticum*	0.0000 [0.0000, 0.0000]	0.0000 [0.0000, 0.0000]	0.0073 [0.0038, 0.0114]	<0.001
	*Clostridium symbiosum*	0.0021 [0.0016, 0.0049]	0.0000 [0.0000, 0.0000]	0.0000 [0.0000, 0.0000]	<0.001
	*Clostridium xylanolyticum*	0.0000 [0.0000, 0.0000]	0.0099 [0.0006, 0.0226]	0.0000 [0.0000, 0.0000]	<0.001
	*Eubacterium hallii*	0.0161 [0.0060, 0.0439]	0.0000 [0.0000, 0.0000]	0.0651 [0.0207, 0.0704]	<0.001
	*Adlercreutzia equolifaciens*	0.0000 [0.0000, 0.0000]	0.0000 [0.0000, 0.0000]	0.0011 [0.0005, 0.0086]	<0.001
	*Alistipes finegoldii *	0.0011 [0.0006, 0.0128]	0.1265 [0.1095, 0.6407]	0.0419 [0.0031, 0.0692]	0.003
	*Alistipes* sp. S457	0.0030 [0.0000, 0.1588]	0.0000 [0.0000, 0.0000]	0.0000 [0.0000, 0.1262]	0.027
	*Alloprevotella rava*	0.0000 [0.0000, 0.0000]	0.0002 [0.0000, 0.0007]	0.0000 [0.0000, 0.0000]	0.007
	*Anaerostipes butyraticus*	0.0000 [0.0000, 0.0000]	0.0000 [0.0000, 0.0000]	0.0220 [0.0031, 0.0334]	<0.001
	*Anaerostipes hadrus*	0.0023 [0.0003, 0.0039]	0.0000 [0.0000, 0.0000]	0.0089 [0.0041, 0.0289]	0.001
	*Anaerotruncus colihominis*	0.0054 [0.0023, 0.0089]	0.0000 [0.0000, 0.0000]	0.0000 [0.0000, 0.0000]	<0.001
	*Anaerotruncus rubiinfantis *	0.0117 [0.0069, 0.0252]	0.0000 [0.0000, 0.0000]	0.0064 [0.0025, 0.0170]	<0.001
	*Angelakisella massiliensis*	0.0212 [0.0065, 0.0532]	0.0000 [0.0000, 0.0000]	0.0000 [0.0000, 0.0000]	<0.001
	*Bacillus nealsonii*	0.0000 [0.0000, 0.0000]	0.0000 [0.0000, 0.0000]	0.0019 [0.0000, 0.0038]	0.003
	*Bacteroides acidifaciens *	0.0158 [0.0116, 0.0317]	0.0000 [0.0000, 0.0000]	0.0265 [0.0254, 0.0307]	<0.001
	*Bacteroides barnesiae*	0.0040 [0.0020, 0.0105]	0.0000 [0.0000, 0.0000]	0.0000 [0.0000, 0.0000]	<0.001
	*Bacteroides caecicola*	0.0000 [0.0000, 0.0007]	0.0000 [0.0000, 0.0000]	0.0000 [0.0000, 0.0000]	0.026
	*Bacteroides clarus *	0.0066 [0.0032, 0.0233]	0.0000 [0.0000, 0.0000]	0.0050 [0.0032, 0.4975]	<0.001
	*Bacteroides faecichinchillae*	0.0000 [0.0000, 0.0000]	0.0000 [0.0000, 0.0000]	0.0094 [0.0031, 0.0176]	<0.001
	*Bacteroides fluxus*	0.0035 [0.0013, 0.0093]	0.0000 [0.0000, 0.0000]	0.0000 [0.0000, 0.0000]	<0.001
	*Bacteroides intestinalis*	0.0000 [0.0000, 0.0000]	0.0000 [0.0000, 0.0000]	0.0018 [0.0005, 0.0046]	<0.001
	*Bacteroides mediterraneensis*	0.0032 [0.0010, 0.1966]	0.0000 [0.0000, 0.0000]	0.0000 [0.0000, 0.0000]	<0.001
	*Bacteroides paurosaccharolyticus*	0.0016 [0.0013, 0.0092]	0.0000 [0.0000, 0.0000]	0.0000 [0.0000, 0.0000]	<0.001
	*Bacteroides rodentium*	0.0000 [0.0000, 0.0000]	0.0000 [0.0000, 0.0000]	0.0005 [0.0003, 0.0010]	<0.001
	*Bacteroides salanitronis*	0.0009 [0.0004, 0.0032]	0.0000 [0.0000, 0.0000]	0.0000 [0.0000, 0.0000]	<0.001
	*Bacteroides salyersiae*	0.0000 [0.0000, 0.0000]	0.0018 [0.0006, 0.0033]	0.0031 [0.0016, 0.2846]	<0.001
	*Bacteroides* sp. 35AE37	0.0000 [0.0000, 0.0000]	0.0000 [0.0000, 0.0000]	0.0022 [0.0011, 0.0031]	<0.001
	*Bacteroides* sp. ANH 2438	0.0002 [0.0000, 0.0013]	0.0000 [0.0000, 0.0000]	0.0011 [0.0005, 0.0023]	0.010
	*Bacteroides* sp. HGA0138	0.0000 [0.0000, 0.0000]	0.0000 [0.0000, 0.0000]	0.0045 [0.0000, 0.0086]	0.003
	*Bacteroides* sp. Marseille P3108	0.0190 [0.0046, 0.0212]	0.0000 [0.0000, 0.0000]	0.0108 [0.0031, 0.0225]	<0.001
	*Bacteroides stercorirosoris*	0.0000 [0.0000, 0.0000]	0.0000 [0.0000, 0.0000]	0.0041 [0.0000, 0.0354]	0.001
	*Bacteroides vulgatus*	0.7618 [0.0572, 1.2351]	1.4728 [0.9327, 2.1484]	1.9609 [1.7974, 3.7528]	0.032
	*Bifidobacterium adolescentis*	0.0000 [0.0000, 0.0000]	0.0000 [0.0000, 0.0000]	0.0011 [0.0000, 0.0099]	0.003
	*Bifidobacterium* sp. 113	0.0000 [0.0000, 0.0000]	0.0012 [0.0004, 0.0197]	0.0000 [0.0000, 0.0000]	<0.001
	*Bifidobacterium* sp. TM 7	0.0000 [0.0000, 0.0000]	0.0000 [0.0000, 0.0005]	0.0000 [0.0000, 0.0000]	0.024
	*Blautia luti *	0.0000 [0.0000, 0.0000]	0.0236 [0.0004, 0.1324]	0.0000 [0.0000, 0.0000]	<0.001
	*Blautia obeum*	0.1137 [0.0579, 0.2103]	0.0627 [0.0455, 0.1708]	0.5450 [0.2173, 0.6162]	0.019
	*Blautia* sp. Canine oral taxon 337	0.0000 [0.0000, 0.0000]	0.0000 [0.0000, 0.0000]	0.0134 [0.0083, 0.0258]	<0.001
	*Butyricicoccus desmolans*	0.0000 [0.0000, 0.0000]	0.0108 [0.0024, 0.0188]	0.0103 [0.0005, 0.0296]	<0.001
	*Butyricimonas faecihominis*	0.0000 [0.0000, 0.0000]	0.0021 [0.0000, 0.0132]	0.0000 [0.0000, 0.0000]	0.007
	*Butyricimonas* sp. 180 3	0.0000 [0.0000, 0.0000]	0.0035 [0.0002, 0.0134]	0.0000 [0.0000, 0.0000]	0.001
	*Butyricimonas* sp. AT11	0.0168 [0.0013, 0.0422]	0.0000 [0.0000, 0.0000]	0.0025 [0.0010, 0.0106]	0.002
	*Butyricimonas* sp. S479	0.0000 [0.0000, 0.0000]	0.0053 [0.0004, 0.0200]	0.0000 [0.0000, 0.0000]	0.001
	*Butyrivibrio crossotus *	0.0000 [0.0000, 0.0000]	0.0023 [0.0014, 0.0029]	0.0000 [0.0000, 0.0000]	<0.001
	*Candidatus Dorea massiliensis*	0.0008 [0.0000, 0.0030]	0.0015 [0.0006, 0.0019]	0.0000 [0.0000, 0.0000]	0.022
	*Candidatus Soleaferrea massiliensis*	0.0019 [0.0010, 0.0069]	0.0000 [0.0000, 0.0000]	0.0000 [0.0000, 0.0000]	<0.001
	*Catabacter hongkongensis*	0.0097 [0.0068, 0.0142]	0.0000 [0.0000, 0.0000]	0.0000 [0.0000, 0.0000]	<0.001
	*Christensenella timonensis*	0.0177 [0.0047, 0.0223]	0.0000 [0.0000, 0.0000]	0.0037 [0.0023, 0.0124]	<0.001
	*Clostridium carnis*	0.0000 [0.0000, 0.0000]	0.0000 [0.0000, 0.0000]	0.0000 [0.0000, 0.0009]	0.044
	*Clostridium phoceensis*	0.0000 [0.0000, 0.0000]	0.0011 [0.0001, 0.0059]	0.0023 [0.0003, 0.0074]	0.003
	*Clostridium* sp. 14,505	0.0000 [0.0000, 0.0000]	0.0000 [0.0000, 0.0006]	0.0000 [0.0000, 0.0000]	0.024
	*Clostridium* sp. 37hoe	0.0034 [0.0000, 0.0135]	0.0000 [0.0000, 0.0000]	0.0114 [0.0062, 0.0294]	0.003
	*Clostridium* sp. ACB 29	0.0000 [0.0000, 0.0000]	0.0000 [0.0000, 0.0000]	0.0011 [0.0005, 0.0035]	<0.001
	*Clostridium* sp. Clone 17	0.0000 [0.0000, 0.0000]	0.0000 [0.0000, 0.0000]	0.0240 [0.0184, 0.0255]	<0.001
	*Clostridium* sp. Culture Jar 19	0.0076 [0.0059, 0.0221]	0.0000 [0.0000, 0.0000]	0.0092 [0.0056, 0.0146]	<0.001
	*Clostridium* sp. Culture 41	0.0056 [0.0044, 0.0072]	0.0000 [0.0000, 0.0000]	0.0062 [0.0022, 0.0092]	<0.001
	*Clostridium* sp. Culture 46	0.0007 [0.0000, 0.0011]	0.0000 [0.0000, 0.0000]	0.0000 [0.0000, 0.0000]	0.001
	*Clostridium* sp. Enrichment culture clone 06 1235251 89	0.0118 [0.0087, 0.0296]	0.0000 [0.0000, 0.0000]	0.0080 [0.0047, 0.0149]	<0.001
	*Clostridium* sp. enrichment culture clone 7 14	0.0007 [0.0000, 0.0088]	0.0000 [0.0000, 0.0000]	0.0043 [0.0023, 0.0097]	0.005
	*Clostridium* sp. Enrichment culture clone VanCtr97	0.0049 [0.0028, 0.0131]	0.0000 [0.0000, 0.0000]	0.0374 [0.0032, 0.0521]	0.001
	*Clostridium* sp. Enrichment culture clone Y234	0.0000 [0.0000, 0.0000]	0.0040 [0.0002, 0.0072]	0.0002 [0.0000, 0.0290]	0.009
	*Clostridium* sp. ID6	0.0000 [0.0000, 0.0000]	0.0000 [0.0000, 0.0000]	0.0054 [0.0000, 0.0073]	0.003
	*Clostridium* sp. Marseille P3122	0.0080 [0.0038, 0.0166]	0.0000 [0.0000, 0.0000]	0.0067 [0.0031, 0.0126]	<0.001
	*Clostridium* sp. Marseille P3244	0.0012 [0.0000, 0.0094]	0.0000 [0.0000, 0.0000]	0.0038 [0.0028, 0.0095]	0.001
	*Clostridium* sp. MLG856.1	0.0000 [0.0000, 0.0089]	0.0000 [0.0000, 0.0000]	0.0000 [0.0000, 0.0000]	0.026
	*Clostridium* sp. PI S10 B5A	0.0000 [0.0000, 0.0000]	0.0000 [0.0000, 0.0000]	0.0000 [0.0000, 0.0003]	0.044
	*Clostridium* sp. TM 40	0.0003 [0.0000, 0.0010]	0.0000 [0.0000, 0.0000]	0.0000 [0.0000, 0.0000]	0.010
	*Clostridium* sp. VKM B 2202	0.0082 [0.0061, 0.0092]	0.0000 [0.0000, 0.0000]	0.0114 [0.0083, 0.0186]	<0.001
	*Coprobacter secundus*	0.0082 [0.0010, 0.0222]	0.0023 [0.0002, 0.0207]	0.0000 [0.0000, 0.0000]	0.010
	*Coprococcus eutactus*	0.0029 [0.0013, 0.0098]	0.0000 [0.0000, 0.0000]	0.0062 [0.0005, 0.0225]	0.003
	*Dakarella massiliensis*	0.1038 [0.0008, 0.7340]	0.0000 [0.0000, 0.0004]	0.0000 [0.0000, 0.0000]	0.002
	*Dialister invisus*	0.0032 [0.0000, 0.0207]	0.0000 [0.0000, 0.0000]	0.0000 [0.0000, 0.0005]	0.015
	*Dialister* sp. GBA27	0.0000 [0.0000, 0.0000]	0.0008 [0.0000, 0.0090]	0.0000 [0.0000, 0.0000]	0.007
	*Dialister* sp. Oral clone MCE7_134	0.0008 [0.0000, 0.0028]	0.0000 [0.0000, 0.0000]	0.0000 [0.0000, 0.0000]	0.010
	*Dialister* sp. oral taxon 119	0.0029 [0.0005, 0.0186]	0.0000 [0.0000, 0.0000]	0.0000 [0.0000, 0.0000]	<0.001
	*Dialister* sp. Oral taxon A97	0.0000 [0.0000, 0.0000]	0.0000 [0.0000, 0.0005]	0.0000 [0.0000, 0.0000]	0.024
	*Dialister succinatiphilus*	0.0024 [0.0000, 0.2609]	0.0038 [0.0015, 0.0074]	0.0000 [0.0000, 0.0000]	0.004
	*Eisenbergiella massiliensis*	0.0000 [0.0000, 0.0014]	0.0061 [0.0013, 0.0322]	0.0000 [0.0000, 0.0000]	0.007
	*Escherichia coli*	0.0019 [0.0009, 0.0237]	0.0000 [0.0000, 0.0000]	0.0011 [0.0000, 0.0073]	0.002
	*Eubacterium coprostanoligenes *	0.0264 [0.0088, 0.0484]	0.0000 [0.0000, 0.0000]	0.1952 [0.0921, 0.6125]	<0.001
	*Faecalibacterium* CM04 06	0.0082 [0.0034, 0.0594]	1.4640 [0.6198, 3.3800]	0.0250 [0.0063, 0.0389]	0.004
	*Faecalibacterium prausnitzii *	3.7151 [2.5044, 5.1455]	12.7134 [10.3494, 14.3182]	5.5894 [3.2108, 7.2897]	<0.001
	*Falcatimonas natans*	0.0000 [0.0000, 0.0000]	0.0021 [0.0001, 0.0042]	0.0086 [0.0009, 0.0434]	0.002
	*Fenollaria timonensis *	0.0126 [0.0048, 0.0157]	0.0000 [0.0000, 0.0000]	0.0181 [0.0107, 0.0238]	<0.001
	*Flintibacter butyricus *	0.0127 [0.0104, 0.0169]	0.0000 [0.0000, 0.0000]	0.0140 [0.0043, 0.0311]	<0.001
	*Holdemania massiliensis*	0.0000 [0.0000, 0.0000]	0.0000 [0.0000, 0.0000]	0.0000 [0.0000, 0.0091]	0.044
	*Howardella ureilytica*	0.0000 [0.0000, 0.0106]	0.0000 [0.0000, 0.0000]	0.0000 [0.0000, 0.0000]	0.026
	*Hungatella hathewayi*	0.0000 [0.0000, 0.0000]	0.0047 [0.0011, 0.0163]	0.0000 [0.0000, 0.0000]	<0.001
	*Intestinibacter bartlettii*	0.0000 [0.0000, 0.0000]	0.0000 [0.0000, 0.0000]	0.0037 [0.0005, 0.0382]	<0.001
	*Intestinimonas butyriciproducens*	0.0000 [0.0000, 0.0000]	0.0021 [0.0010, 0.0056]	0.0000 [0.0000, 0.0000]	<0.001
	*Intestinimonas gabonensis*	0.0108 [0.0077, 0.0270]	0.0925 [0.0581, 0.1601]	0.0067 [0.0023, 0.0165]	<0.001
	*Intestinimonas timonensis*	0.0031 [0.0017, 0.0180]	0.0000 [0.0000, 0.0000]	0.0000 [0.0000, 0.0000]	<0.001
	*Lachnobacterium bovis*	0.0000 [0.0000, 0.0000]	0.0000 [0.0000, 0.0000]	0.0152 [0.0011, 0.0187]	<0.001
	*Mediterranea massiliensis*	0.0009 [0.0003, 0.0014]	0.0000 [0.0000, 0.0000]	0.0000 [0.0000, 0.0000]	<0.001
	*Megasphaera elsdenii*	0.0004 [0.0000, 0.0077]	0.0000 [0.0000, 0.0000]	0.0000 [0.0000, 0.0000]	0.010
	*Merdibacter massiliensis*	0.0026 [0.0009, 0.0055]	0.0000 [0.0000, 0.0000]	0.0000 [0.0000, 0.0000]	<0.001
	*Methanobrevibacter smithii*	0.0061 [0.0000, 0.0322]	0.0019 [0.0000, 0.0329]	0.0000 [0.0000, 0.0000]	0.031
	*Oribacterium* sp. NK2B42	0.0033 [0.0011, 0.0091]	0.0000 [0.0000, 0.0000]	0.0000 [0.0000, 0.0000]	<0.001
	*Oxalobacter formigenes*	0.0000 [0.0000, 0.0000]	0.0000 [0.0000, 0.0000]	0.0172 [0.0005, 0.0280]	<0.001
	*Pantoea dispersa*	0.0013 [0.0004, 0.0102]	0.0000 [0.0000, 0.0000]	0.0000 [0.0000, 0.0000]	<0.001
	*Papillibacter cinnamivorans*	0.0000 [0.0000, 0.0000]	0.0083 [0.0024, 0.0115]	0.0057 [0.0002, 0.0086]	<0.001
	*Parabacteroides merdae*	0.0007 [0.0000, 0.0013]	0.0004 [0.0000, 0.0056]	0.0000 [0.0000, 0.0000]	0.025
	*Parabacteroides* sp. SN4	0.0000 [0.0000, 0.0034]	0.0000 [0.0000, 0.0000]	0.0000 [0.0000, 0.0000]	0.026
	*Phascolarctobacterium* sp. 377	0.0000 [0.0000, 0.0000]	0.0000 [0.0000, 0.0000]	0.0000 [0.0000, 0.0034]	0.012
	*Phascolarctobacterium succinatutens*	0.0000 [0.0000, 0.0000]	0.0002 [0.0000, 1.0265]	0.0000 [0.0000, 0.0023]	0.050
	*Phocea massiliensis*	0.0031 [0.0020, 0.0116]	0.0000 [0.0000, 0.0000]	0.0000 [0.0000, 0.0000]	<0.001
	*Prevotella copri*	0.0009 [0.0000, 0.0112]	0.0000 [0.0000, 0.0000]	0.0000 [0.0000, 0.0000]	0.001
	*Prevotella* sp. 109	0.0048 [0.0003, 1.4759]	0.0000 [0.0000, 0.0000]	0.0034 [0.0022, 0.0074]	0.005
	*Prevotella* sp. Canine oral taxon 282	0.0008 [0.0004, 0.0011]	0.0000 [0.0000, 0.0000]	0.0000 [0.0000, 0.0000]	<0.001
	*Prevotella* sp. DJF_RP53	0.0000 [0.0000, 0.0003]	0.0029 [0.0022, 0.0957]	0.0000 [0.0000, 0.0000]	<0.001
	*Prevotella stercorea*	0.0002 [0.0000, 0.0064]	0.0006 [0.0001, 0.0034]	0.0000 [0.0000, 0.0000]	0.034
	*Propionibacterium* sp. S342	0.1322 [0.0024, 0.1969]	0.0000 [0.0000, 0.0000]	0.1538 [0.0062, 0.6465]	<0.001
	*Pseudoflavonifractor* sp. Marseille P3106	0.0000 [0.0000, 0.0000]	0.0000 [0.0000, 0.0000]	0.0023 [0.0012, 0.0050]	<0.001
	*Robinsoniella peoriensis*	0.0000 [0.0000, 0.0000]	0.0000 [0.0000, 0.0000]	0.0046 [0.0032, 0.0138]	<0.001
	*Robinsoniella* sp. MCWD5	0.0110 [0.0042, 0.0156]	0.0000 [0.0000, 0.0000]	0.0298 [0.0276, 0.0405]	<0.001
	*Roseburia inulinivorans*	0.0639 [0.0282, 0.1109]	0.5866 [0.1126, 1.7230]	0.0204 [0.0002, 0.0274]	0.006
	*Roseburia* sp. 831b	0.0672 [0.0000, 0.4704]	0.9504 [0.2563, 1.5792]	0.0000 [0.0000, 0.0003]	0.030
	*Roseburia* sp. DJF_VR77	0.0000 [0.0000, 0.0000]	0.0000 [0.0000, 0.0000]	0.0026 [0.0000, 0.0345]	0.003
	*Ruminiclostridium thermocellum*	0.0000 [0.0000, 0.0000]	0.0000 [0.0000, 0.0000]	0.0021 [0.0014, 0.0045]	<0.001
	*Ruminococcus flavefaciens*	0.0090 [0.0010, 0.0314]	0.0063 [0.0025, 0.0110]	0.0000 [0.0000, 0.0000]	0.001
	*Ruminococcus* sp. 16442	0.0068 [0.0053, 0.0099]	0.0000 [0.0000, 0.0000]	0.0113 [0.0002, 0.0147]	0.001
	*Ruminococcus* sp. 653	0.0000 [0.0000, 0.0000]	0.0103 [0.0074, 0.0152]	0.0140 [0.0070, 0.0260]	<0.001
	*Ruminococcus* sp. AT10	0.0000 [0.0000, 0.0000]	0.0000 [0.0000, 0.0000]	0.0092 [0.0037, 0.1247]	<0.001
	*Ruminococcus* sp. DJF_VR52	0.0000 [0.0000, 0.0000]	0.0000 [0.0000, 0.0000]	0.0005 [0.0000, 0.0034]	0.003
	*Ruminococcus* sp. DJF_VR70k1	0.0000 [0.0000, 0.0000]	0.0040 [0.0008, 0.0102]	0.0000 [0.0000, 0.0000]	<0.001
	*Ruminococcus* sp. ID1	0.0235 [0.0164, 0.0259]	0.0000 [0.0000, 0.0000]	0.0368 [0.0172, 0.0697]	<0.001
	*Ruminococcus* sp. ZS2 15	0.0000 [0.0000, 0.0000]	0.0000 [0.0000, 0.0000]	0.0011 [0.0005, 0.0063]	<0.001
	*Saccharofermentans acetigenes*	0.0099 [0.0047, 0.0158]	0.0000 [0.0000, 0.0000]	0.0000 [0.0000, 0.0000]	<0.001
	*Streptococcus agalactiae*	0.0000 [0.0000, 0.0000]	0.0000 [0.0000, 0.0000]	0.0000 [0.0000, 0.0005]	0.012
	*Streptococcus equinus*	0.0000 [0.0000, 0.0000]	0.0000 [0.0000, 0.0000]	0.0035 [0.0013, 0.0161]	<0.001
	*Streptococcus mitis*	0.0000 [0.0000, 0.0009]	0.0000 [0.0000, 0.0000]	0.0000 [0.0000, 0.0000]	0.026
	*Succinivibrio dextrinosolvens*	0.0008 [0.0000, 0.0012]	0.0003 [0.0000, 0.0004]	0.0000 [0.0000, 0.0000]	0.030
	*Sutterella* sp. 252	0.0000 [0.0000, 0.0843]	0.0000 [0.0000, 0.0000]	0.0000 [0.0000, 0.0000]	0.026
	*Tyzzerella* sp. Marseille P3062	0.0000 [0.0000, 0.0000]	0.0000 [0.0000, 0.0000]	0.0002 [0.0000, 0.0057]	0.003

^#^ KW: *p*-value from Kruskal-Wallis test.

## Supplementary Materials

The following are available online at https://www.mdpi.com/1660-4601/16/21/4065/s1, Figure S1: Rarefaction curve revealing the number of identified OTUs as a function of the number of reads by regions’ group.

## References

[1] Schwabe R.F., Jobin C. (2013). The microbiome and cancer. Nat. Rev. Cancer.

[2] O’Hara A.M., Shanahan F. (2006). The gut flora as a forgotten organ. EMBO Rep..

[3] García-Castillo V., Sanhueza E., McNerney E., Onate S.A., García A. (2016). Microbiota dysbiosis: A new piece in the understanding of the carcinogenesis puzzle. J. Med Microbiol..

[4] David L.A., Maurice C.F., Carmody R.N., Gootenberg D.B., Button J.E., Wolfe B.E., Ling A.V., Devlin A.S., Varma Y., Fischbach M.A. (2014). Diet rapidly and reproducibly alters the human gut microbiome. Nature.

[5] Gupta V.K., Paul S., Dutta C. (2017). Geography, Ethnicity or Subsistence-Specific Variations in Human Microbiome Composition and Diversity. Front. Microbiol..

[6] Eckburg P.B., Bik E.M., Bernstein C.N., Purdom E., Dethlefsen L., Sargent M., Gill S.R., Nelson K.E., Relman D.A. (2005). Diversity of the human intestinal microbial flora. Science.

[7] Turnbaugh P.J., Hamady M., Yatsunenko T., Cantarel B.L., Duncan A., Ley R.E., Sogin M.L., Jones W.J., Roe B.A., Affourtit J.P. (2009). A core gut microbiome in obese and lean twins. Nature.

[8] Armougom F., Henry M., Vialettes B., Raccah D., Raoult D. (2009). Monitoring Bacterial Community of Human Gut Microbiota Reveals an Increase in Lactobacillus in Obese Patients and Methanogens in Anorexic Patients. PLoS ONE.

[9] Sanz Y., Romani-Perez M., Benitez-Paez A., Portune K.J., Brigidi P., Rampelli S., Dinan T., Stanton C., Delzenne N., Blachier F. (2018). Towards microbiome-informed dietary recommendations for promoting metabolic and mental health: Opinion papers of the MyNewGut project. Clin. Nutr..

[10] Iebba V., Totino V., Gagliardi A., Santangelo F., Cacciotti F., Trancassini M., Mancini C., Cicerone C., Corazziari E., Pantanella F. (2016). Eubiosis and dysbiosis: The two sides of the microbiota. New Microbiol..

[11] Arumugam M., Raes J., Pelletier E., Le Paslier D., Yamada T., Mende D.R., Fernandes G.R., Tap J., Bruls T., Batto J.-M. (2011). Enterotypes of the human gut microbiome. Nature.

[12] Conlon M.A., Bird A.R. (2014). The impact of diet and lifestyle on gut microbiota and human health. Nutrients.

[13] Mueller S., Saunier K., Hanisch C., Norin E., Alm L., Midtvedt T., Cresci A., Silvi S., Orpianesi C., Verdenelli M.C. (2006). Differences in Fecal Microbiota in Different European Study Populations in Relation to Age, Gender, and Country: A Cross-Sectional Study. Appl. Environ. Microbiol..

[14] Shin J.-H., Sim M., Lee J.-Y., Shin D.-M. (2016). Lifestyle and geographic insights into the distinct gut microbiota in elderly women from two different geographic locations. J. Physiol. Anthr..

[15] Yilmaz B., Spalinger M.R., Biedermann L., Franc Y., Fournier N., Rossel J.-B., Juillerat P., Rogler G., MacPherson A.J., Scharl M. (2018). The presence of genetic risk variants within PTPN2 and PTPN22 is associated with intestinal microbiota alterations in Swiss IBD cohort patients. PLoS ONE.

[16] De Filippo C., Di Paola M., Ramazzotti M., Albanese D., Pieraccini G., Banci E., Miglietta F., Cavalieri D., Lionetti P. (2017). Diet, Environments, and Gut Microbiota. A Preliminary Investigation in Children Living in Rural and Urban Burkina Faso and Italy. Front. Microbiol..

[17] Senghor B., Sokhna C., Ruimy R., Lagier J.-C. (2018). Gut microbiota diversity according to dietary habits and geographical provenance. Hum. Microbiome J..

[18] Suzuki T.A., Worobey M. (2014). Geographical variation of human gut microbial composition. Boil. Lett..

[19] Yatsunenko T., Rey F.E., Manary M.J., Trehan I., Dominguez-Bello M.G., Contreras M., Magris M., Hidalgo G., Baldassano R.N., Anokhin A.P. (2012). Human gut microbiome viewed across age and geography. Nature.

[20] Tyakht A.V., Kostryukova E.S., Popenko A.S., Belenikin M.S., Pavlenko A.V., Larin A.K., Karpova I.Y., Selezneva O.V., Semashko T.A., Ospanova E.A. (2013). Human gut microbiota community structures in urban and rural populations in Russia. Nat. Commun..

[21] He Y., Wu W., Zheng H.-M., Li P., McDonald D., Sheng H.-F., Chen M.-X., Chen Z.-H., Ji G.-Y., Zheng Z.-D.-X. (2018). Author Correction: Regional variation limits applications of healthy gut microbiome reference ranges and disease models. Nat. Med..

[22] Panebianco C., Potenza A., Andriulli A., Pazienza V. (2018). Exploring the microbiota to better understand gastrointestinal cancers physiology. Clin. Chem. Lab. Med..

[23] Panebianco C., Andriulli A., Pazienza V. (2018). Pharmacomicrobiomics: Exploiting the drug-microbiota interactions in anticancer therapies. Microbiome.

[24] Rajpoot M., Sharma A.K., Sharma A., Gupta G.K. (2018). Understanding the microbiome: Emerging biomarkers for exploiting the microbiota for personalized medicine against cancer. Semin. Cancer Boil..

[25] Wang B., Yao M., Lv L., Ling Z., Li L. (2017). Th Human Microbiota in Health and Disease. Engineering.

[26] Zhou Y., Xu Z.Z., He Y., Yang Y., Liu L., Lin Q., Nie Y., Li M., Zhi F., Liu S. (2018). Gut Microbiota Offers Universal Biomarkers across Ethnicity in Inflammatory Bowel Disease Diagnosis and Infliximab Response Prediction. MSystems.

[27] Picchianti-Diamanti A., Panebianco C., Salemi S., Sorgi M.L., Di Rosa R., Tropea A., Sgrulletti M., Salerno G., Terracciano F., D’Amelio R. (2018). Analysis of Gut Microbiota in Rheumatoid Arthritis Patients: Disease-Related Dysbiosis and Modifications Induced by Etanercept. Int. J. Mol. Sci..

[28] Langille M.G.I., Zaneveld J., Caporaso J.G., McDonald D., Knights D., Reyes J.A., Clemente J.C., Burkepile D.E., Thurber R.L.V., Knight R. (2013). Predictive functional profiling of microbial communities using 16S rRNA marker gene sequences. Nat. Biotechnol..

[29] Odamaki T., Kato K., Sugahara H., Hashikura N., Takahashi S., Xiao J.-Z., Abe F., Osawa R. (2016). Age-related changes in gut microbiota composition from newborn to centenarian: A cross-sectional study. BMC Microbiol..

[30] Biagi E., Nylund L., Candela M., Ostan R., Bucci L., Pini E., Nikkilä J., Monti D., Satokari R., Franceschi C. (2010). Correction: Through Ageing, and Beyond: Gut Microbiota and Inflammatory Status in Seniors and Centenarians. PLoS ONE.

[31] Faith J.J., Guruge J.L., Charbonneau M., Subramanian S., Seedorf H., Goodman A.L., Clemente J.C., Knight R., Heath A.C., Leibel R.L. (2013). The long-term stability of the human gut microbiota. Science.

[32] Rodriguez J.M., Murphy K., Stanton C., Ross R.P., Kober O.I., Juge N., Avershina E., Rudi K., Narbad A., Jenmalm M.C. (2015). The composition of the gut microbiota throughout life, with an emphasis on early life. Microb. Ecol. Health Dis..

[33] Das B., Ghosh T.S., Kedia S., Rampal R., Saxena S., Bag S., Mitra R., Dayal M., Mehta O., Surendranath A. (2018). Analysis of the Gut Microbiome of Rural and Urban Healthy Indians Living in Sea Level and High Altitude Areas. Sci. Rep..

[34] Costello E.K., Stagaman K., Dethlefsen L., Bohannan B.J.M., Relman D.A. (2012). The application of ecological theory toward an understanding of the human microbiome. Science.

[35] McCarville J.L., Caminero A., Verdu E.F. (2016). Novel perspectives on therapeutic modulation of the gut microbiota. Ther. Adv. Gastroenterol..

[36] Gaulke C.A., Sharpton T.J. (2018). The influence of ethnicity and geography on human gut microbiome composition. Nat. Med..

