# Supplementary figures and images for: Gut Microbiota Profiles Differ among Individuals Depending on Their Region of Origin: An Italian Pilot Study
Source: Int J Environ Res Public Health. 2019 Oct 23;16(21):4065. doi: 10.3390/ijerph16214065 (PMC6862301; doi:10.3390/ijerph16214065)

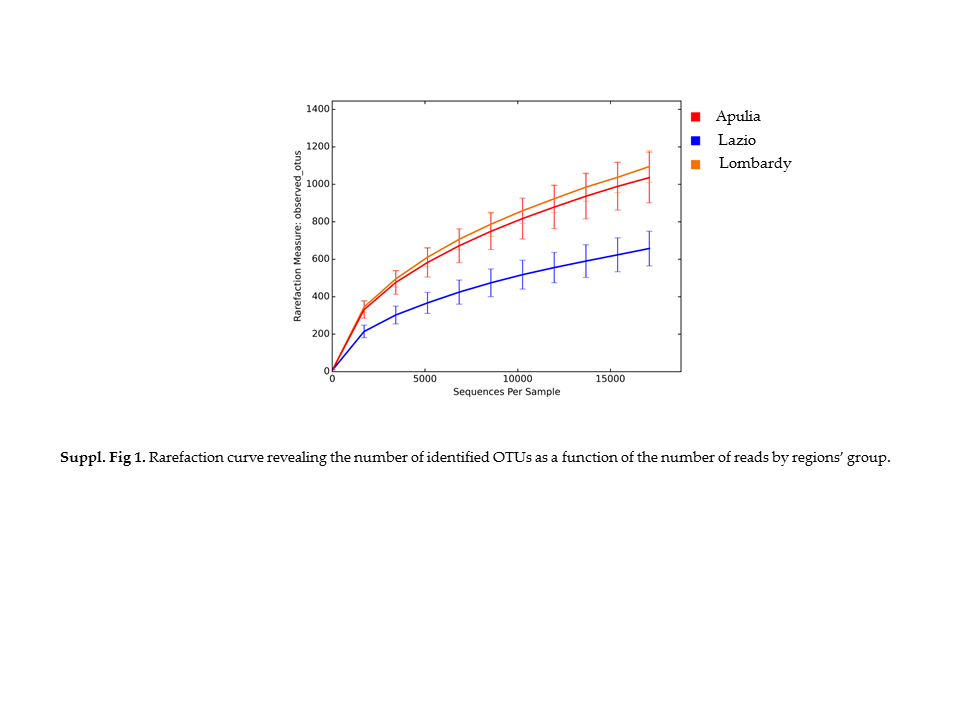

Supplement: Supplementary file 1 [file ijerph-16-04065-s001.zip › ijerph-606796-supplementary.tif]
